# Is there a requirement for advanced airway management for trauma patients in the pre-hospital phase of care?

**DOI:** 10.1186/1757-7241-22-S1-P8

**Published:** 2014-07-07

**Authors:** David J Lockey, Beth A Healey, Anne E Weaver, Graham Chalk, Gareth E Davies

**Affiliations:** 1 Londons Air Ambulance, Royal London Hospital, London E1 1BB, UK

## Background

This study was performed to establish the frequency of pre-hospital airway compromise in trauma patients attended by a UK physician-led pre-hospital service and the role of advanced airway interventions in the treatment of the compromise.

## Method

Over a one year period (April 2012-March 2013) all pre-hospital trauma patients attended by the doctor-paramedic LAA team who were identified as having airway compromise or an indication for any airway intervention on scene were included in a prospective observational study. The doctor paramedic team recorded any airway compromise on arrival and prior interventions carried out by ambulance service personnel.

## Results

472 patients met the inclusion criteria. Ambulance service personnel attended before the enhanced care team in 469/472 (99.4%) of cases. 200 patients had no evidence of compromise of which 134 (67%) had received airway interventions by the ambulance service. 269 (57%) had airway compromise at the point of arrival of the enhanced care team. Of the 269 with airway compromise, 174 had complete or partial airway obstruction and 145 had gross contamination with blood or vomit. Ninety-eight patients received paramedic advanced airway management interventions (intubation without drugs or supraglottic airway insertion). 48/50 (92%) supraglottic airway insertions and 29/45 (64%) intubations were successful. All patients had successful pre-hospital intubation by the enhanced care team before transport to hospital.

## Discussion

The reported results suggest a requirement for on-scene advanced airway management in a relatively small number of severely injured trauma patients. Standard ambulance service interventions do not appear to adequately treat airway compromise in a small proportion of trauma patients. Intubation without drugs had a high failure rate. These results suggest that the airway management problems reported in the NCEPOD report: Trauma who cares? in 2007 still exist.
